# Inflammatory markers as predictors of severity in adult incarcerated groin hernias – a retrospective comparative study

**DOI:** 10.1186/s12893-026-03498-8

**Published:** 2026-01-17

**Authors:** Xigui Tian, Caihao Tang, Jiaming Lan

**Affiliations:** Department of Gastrointestinal Hernia Surgery, Liangping District People’s Hospital, Chongqing, No. 16, Bigui Road, Shuanggui Street, Liangping District, Chongqing, 405200 China

**Keywords:** Incarcerated groin hernia, Bowel resection, Inflammatory markers

## Abstract

**Background:**

Diagnosing incarcerated groin hernia and predicting its progression to strangulation remains challenging. This study investigates whether blood inflammatory markers can aid in diagnosing incarcerated groin hernias and assessing their severity.

**Methods:**

A retrospective analysis was conducted on patients who underwent surgery for incarcerated groin hernia between 2018 and 2024. Preoperative blood tests were performed, and patients were categorized into bowel resection and non-resection groups.

**Results:**

Among 203 patients, 78 required bowel resection. Significant differences were observed in hernia type, white blood cell count, neutrophil percentage, C-reactive protein, neutrophil-to-lymphocyte ratio, and serum Sodium-ion levels between the two groups.

**Conclusion:**

White blood cell count, neutrophil percentage, neutrophil-to-lymphocyte ratio, and C-reactive protein are effective diagnostic markers for incarcerated groin hernia. Combining these inflammatory markers provides a reliable method for predicting disease severity.

## Introduction

Groin hernia is a common surgical disease, and for adult patients, surgical treatment is the most effective treatment method [1]. As the most common elective surgery worldwide, over 20 million people undergo groin hernia surgery every year for treatment [2]. However, when the abdominal organs enter the hernia sac and cannot self-reduce due to the narrowing of the outer ring, they remain inside the hernia sac, leading to disturbance of blood circulation, which is called incarcerated groin hernia. This is a disease that requires emergency surgery, with approximately 5–15% of patients with groin hernia experiencing incarceration [2–4].

Incarcerated groin hernia is a common cause of intestinal obstruction [5]. If the incarcerated groin hernia can be relieved in time, the affected intestinal tract can return to normal, and intestinal obstruction can be cured. If the incarceration cannot be relieved in a timely manner, the increasing pressure on the intestinal tract and mesentery can reduce arterial blood flow, ultimately leading to complete blockage and the formation of strangulated groin hernia. At this point, the pulsation of the mesenteric artery disappears, and the intestinal wall gradually loses its luster, elasticity, and peristaltic ability, ultimately leading to intestinal necrosis. The hospitalization time of patients with intestinal necrosis will be significantly increased, and postoperative complications will be higher, about 6% −43% [6–9], with a mortality rate of 1% −7% [2, 7, 10].

When the intestinal blood supply of incarcerated groin hernia is good, manual reduction is an acceptable treatment option, especially for patients with high anesthesia risk [3]. Exploratory laparotomy and laparoscopic exploration are common surgical methods for incarcerated groin hernia. If preoperative judgment requires intestinal resection, it will affect the choice of surgical method by the surgeon [11]. Therefore, preoperative assessment of whether intestinal ischemic necrosis has occurred in incarcerated groin hernia is helpful for clinical doctors in diagnosis and treatment selection. For example, advanced age, female gender, and type of groin hernia are common high-risk factors for intestinal resection of incarcerated groin hernia [5, 12, 13]. Given the well-established role of inflammatory markers in diagnosing and assessing the severity of acute abdominal conditions such as appendicitis [14–16], we hypothesized that they might similarly be useful in predicting the severity of incarcerated groin hernias. This retrospective study aims to predict the severity of incarcerated groin hernia by analyzing different inflammatory markers.

## Material and methods

### Patients and data collection

Patients diagnosed with incarcerated groin hernia and undergoing surgical treatment in our hospital will be selected. We retrieved hospitalized patients related to the period from January 1, 2018 to December 31, 2024. This study has obtained the approval of the Ethics Committee of Liangping District People’s Hospital in Chongqing.

All patients diagnosed with incarcerated groin hernia will be selected by us, and we will confirm the diagnosis based on surgical records. The patient selection was based on the following criteria:

- Inclusion Criteria:
  - Adult patients (age > 18 years).
  - Be treated surgically.
  - Did not receive antibiotic treatment prior to hospital admission.
- Exclusion Criteria:
  - Successful manual reduction and scheduled surgery.
  - The contents of the incarcerated hernia are the omentum.
  - Not receiving surgical treatment within 12 h of admission.
  - Incarcerated groin hernia that occurred during hospitalization.
  - Have blood system diseases and other infectious diseases.

All data comes from the patient’s medical records, and we collected the following data: age, gender, month of onset, the time interval between symptom onset and hospitalization, the time interval between hospitalization and surgery, preoperative white blood cell count (WBCC), percentage of neutrophils (NE%), C-reactive protein (CRP), neutrophil/lymphocyte ratio (NLR), serum Sodium-ion(Na^+^), type of hernia, hernia side, incarcerated organ, recurrent hernia, bowel resection.

At our institution, patients will have their blood samples drawn upon admission. And sudden irreversible mass in the inguinal region accompanied by pain is defined as the time of symptom onset. We will designate March 21 st to September 22nd as spring and summer, and September 23rd to March 20th of the following year as autumn and winter. When the patient is diagnosed with incarcerated groin hernia and there are no signs of peritonitis, we will perform manual reduction with the patient’s informed consent. If the manual reset fails, we will perform emergency surgery on the patient.

Blood samples are measured by an automated hematology analyzer (Sysmex NX-10) and a fully automated electrolyte analyzer (LABOSPECT 008). In our institution, we consider WBCC, NE%, and CRP as common inflammatory markers, with abnormal reference upper limits of 9.5*10^9^/L, 75.0%, and 10.0 mg/L, respectively. We found that NLR, as an inflammatory marker, can also be used as a laboratory auxiliary marker for the diagnosis of incarcerated groin hernia [5, 17, 18]. The reference lower limit of serum Na^+^ is 137.0 mmol/L. In this study, the primary indicator of disease severity for an incarcerated groin hernia was defined as the intraoperative finding that necessitated a bowel resection. We decided whether to perform intestinal resection based on intraoperative exploration of intestinal ischemia, and divided the patients into two groups: bowel resection group and no bowel resection group. We explore sensitive indicators of intestinal necrosis by analyzing and comparing data from two groups of patients.

### Statistical analysis

We used SPSS 24.0 (IBM, Armonk, NY, USA) software to analyze all the research data. Categorical variables are analyzed using percentages for descriptive statistics, and Chi-square test or Fisher’s exact tests are used for analysis depending on the situation. Continuous variables with normal distribution are represented by mean ± (standard deviation), maximum and minimum values, and then independent sample t-test is used to detect whether there is a difference between the two groups.

We use ROC curve to determine the optimal critical value of inflammatory markers for intestinal necrosis. The maximum value of the Youden index corresponds to the optimal diagnostic threshold, which is the cutoff value. We will calculate the area under the curve (AUC), ROC curve, and 95% confidence interval (CI) of AUC. In our analysis process, *p* < 0.05 will be considered statistically significant.

## Results

According to the inclusion and exclusion criteria, a total of 203 patients were selected for this study. Among them, there were more male patients than female patients, with 117 male patients (57.6%) and 86 female patients (42.4%). Some factors (categorical variables) that may affect intestinal resection for incarcerated groin hernia are presented in Table 1. There were 78 patients in the intestinal resection group, including 42 male patients (53.8%) and 36 female patients 36(46.2%). There were 125 patients in the non-intestinal resection group, including 75 male patients (60.0%) and 50 female patients (40.0%). There is no statistical difference between these two groups (*p* = 0.388). There was no statistical difference between the two groups in terms of the season of onset, previous occurrence of groin hernia, hernia side, incarcerated organ, and time from admission to surgery (*p* = 0.798, *p* = 0.748, *p* = 0.122, *p* = 0.078, *p* = 0.260). Type of hernia, WBCC, NE%, CRP, NLR, and serum Na + showed statistical differences between the two groups (*p* < 0.05). In the intestinal resection group, there were 56 cases of femoral hernia patients, accounting for a relatively high proportion of 71.8%. In the non-intestinal resection group, there were 70 cases of indirect hernia patients, accounting for a relatively high proportion of 56.0%.

**Table 1:** Univariate analysis of factors affecting intestinal resection for incarcerated groin hernia (categorical variables)

Characteristics	Bowel resection *n* = 78	No bowel resection *n* = 125	*P* value	Total *n* = 203
Sex				
Male	42(53.8%)	75(60.0%)		117(57.6%)
Female	36(46.2%)	50(40.0%)	0.388	86(42.4%)
Admission season				
Spring and summer	36(46.2%)	60(48.0%)		96(47.3%)
Autumn and winter	42(53.8%)	65(52.0%)	0.798	107(52.7%)
Type of hernia				
Indirect hernia	22(28.2%)	70(56.0%)		92(45.3%)
Femoral hernia	56(71.8%)	50(40.0%)	< 0.001	106(52.2%)
Direct hernia	0(0.0%)	5(4.0%)		5(2.5%)
Recurrent hernia				
Yes	3(3.8%)	6(4.8%)		9(4.4%)
No	75(96.2%)	119(95.2%)	0.748	194(95.6%)
Hernia side				
Left	27(34.6%)	57(45.6%)		84(41.4%)
Right	51(65.4%)	68(54.4%)	0.122	119(58.6%)
Incarcerated organ				
Small bowel	76(97.4%)	114(91.2%)		190(93.6%)
Colon	2(2.6%)	11(8.8%)	0.078	13(6.4%)
WBCC (10^9^/L)				
≤ 9.5	39(50.0%)	88(70.4%)		127(62.6%)
> 9.5	39(50.0%)	37(29.6%)	0.003	76(37.4%)
NE%				
≤ 75.0	7(9.0%)	59(47.2%)		66(32.5%)
> 75.0	71(91.0%)	66(52.8%)	< 0.001	137(67.5%)
CRP (mg/L)				
≤ 10.0	25(32.1%)	110(88.0%)		135(66.5%)
> 10	53(67.9%)	15(12.0%)	< 0.001	68(33.5%)
NLR				
≤ 8.2	24(30.8%)	94(75.2%)		118(58.1%)
> 8.2	54(69.2%)	31(24.8%)	< 0.001	85(41.9%)
Na^+^ (mmol/L)				
≤ 137.0	18(23.1%)	5(4.0%)		23(11.3%)
> 137.0	60(76.9%)	120(96.0%)	< 0.001	180(88.7%)
In-hospital time (h)				
≤ 6.0	47(60.3%)	85(68.0%)		132(65.0%)
> 6.0	31(39.7%)	40(32.0%)	0.260	71(35.0%)

*WBCC* preoperative white blood cell count, *NE%* percentage of neutrophils, *CRP* C-reactive protein, *NLR* neutrophil/lymphocyte ratio

Univariate analysis of factors affecting intestinal resection for incarcerated groin hernia (continuous variable) is shown in Table 2. The average age of the intestinal resection group was 72.90 ± 8.33 years, while the average age of the non-intestinal resection group was 68.08 ± 11.74 years. The age of the intestinal resection group is higher than that of the non-intestinal resection group, and there is a statistical difference between them (*p* < 0.05). There were also statistical differences in duration of incarceration, WBCC, NE%, CRP, and NLR between the two groups (*p* < 0.05). The duration of incarceration, WBCC, NE%, CRP, and NLR values of the intestinal resection group were significantly higher than those of the non-intestinal resection group. Finally, we can observe that the range of maximum and minimum values in each group is quite large.

**Table 2:** Univariate analysis of factors affecting intestinal resection for incarcerated groin hernia (continuous variable)

Characteristics	Bowel resection *n* = 78	No bowel resection *n* = 125	*P* value
Age (years, mean ± SD, (min, max))	72.90 ± 8.33(46,88)	68.08 ± 11.74(35,92)	0.002
Duration of incarceration (h, mean ± SD, (min, max))	62.10 ± 58.50(3,288)	33.12 ± 48.49(2,240)	< 0.001
WBCC (10^9^/L, mean ± SD, (min, max))	9.81 ± 3.33(2.41,15.87)	8.11 ± 3.04(2.95,17.26)	< 0.001
NE% (mean ± SD, (min, max))	83.64 ± 6.70(65.7,95.2)	75.16 ± 10.37(54.1,93.7)	< 0.001
NLR (mean ± SD, (min, max))	12.30 ± 8.15(2.88,40.56)	7.03 ± 5.83(1.58,32.34)	< 0.001
CRP (mg/L, mean ± SD, (min, max))	43.63 ± 44.11(10,175.57)	13.37 ± 12.54(10,96.91)	< 0.001

*SD* standard deviation

The sensitivity of inflammatory markers in the diagnosis of whether to perform intestinal resection based on surgical results is shown in Table 3. The increase of individual inflammatory markers, such as WBCC, NE%, CRP, and NLR, has higher sensitivity in the intestinal resection group, but lower sensitivity in the non-intestinal resection group. And we analyzed the situation where several inflammatory markers were either normal or elevated simultaneously. When several inflammatory markers are normal at the same time, it has extremely high sensitivity for diagnosing patients who have not undergone intestinal resection surgery. When the values of several inflammatory markers increase simultaneously, it has extremely high sensitivity for diagnosing patients undergoing intestinal resection surgery. It is worth mentioning that even if inflammatory markers are normal or elevated at the same time, it cannot accurately predict whether intestinal resection surgery will be performed.

**Table 3:** According to the surgical results, the sensitivity of inflammatory markers (WBCC, NE%, NLR, and CRP) to elevated or simultaneously elevated or simultaneously normal is determined

	Bowel resection *n* = 78	No bowel resection *n* = 125	Total *n* = 203
Elevated WBCC	39(50.0%)	37(29.6%)	76(37.4%)
Elevated NE%	71(91.0%)	66(52.8%)	137(67.5%)
Elevated NLR	54(69.2%)	31(24.8%)	85(41.9%)
Elevated CRP	53(67.9%)	15(12.0%)	68(33.5%)
Normal WBCC, NE%, CRP	3(3.8%)	45(36.0%)	48(23.6%)
Normal WBCC, NE%, NLR, CRP	2(2.6%)	45(36.0%)	47(23.2%)
Elevated WBCC, NE%, CRP	30(38.5%)	3(2.4%)	33(16.3%)
Elevated WBCC, NE%, NLR, CRP	27(34.6%)	1(0.8%)	28(13.8%)

**Fig. 1:**
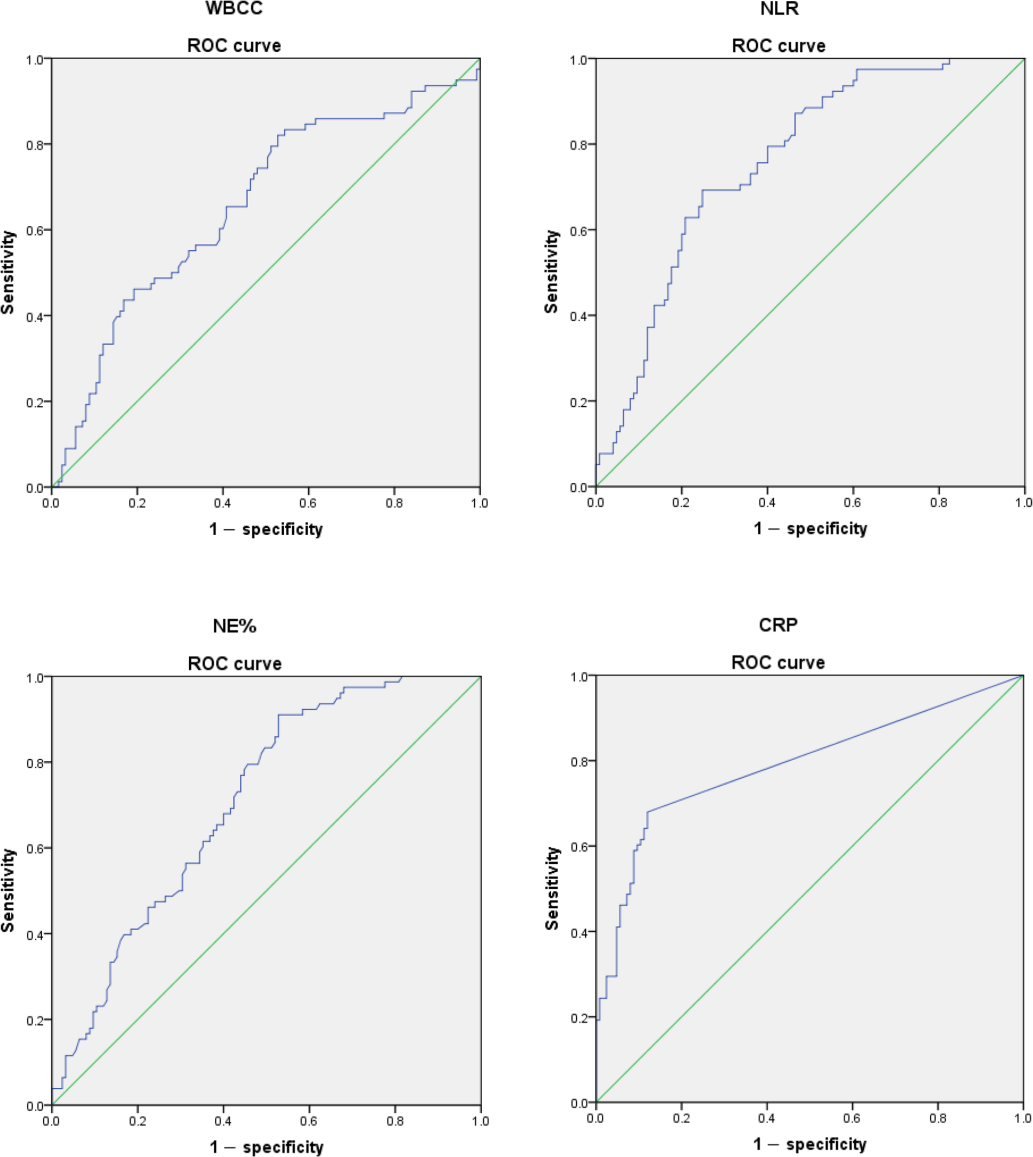
ROC analysis for preoperative white blood cell count (WBCC), percentage of neutrophils (NE%), neutrophil/lymphocyte ratio (NLR) and C-reactive protein (CRP)

The ROC curve analysis results of four inflammatory markers are shown in Fig. 1. The ROC curve analysis results show that WBCC, NE%, CRP, and NLR have good predictive effects on intestinal necrosis (AUC = 0.659,0.708,0.761,0.790).

The ROC curve analysis results of inflammatory markers are shown in Table 4. The cutoff value of WBCC is 7.3 * 10^9^/L, with a sensitivity of 82.1% and a specificity of 47.2% (AUC = 0.659; 95% CI, 0.580–0.737; *p* < 0.001). The cutoff value of NE% is 75.3%, the sensitivity is 91.0%, and the specificity is 47.2% (AUC = 0.708; 95% CI, 0.638–0.778; *p* < 0.001). The cutoff value of NLR is 8.2, with a sensitivity of 69.2% and a specificity of 75.2% (AUC = 0.761; 95% CI, 0.696–0.826; *p* < 0.001). The cutoff value of CRP is 10.0 mg/L, with a sensitivity of 67.9% and a specificity of 88.0% (AUC = 0.790; 95% CI, 0.720–0.859; *p* < 0.001).

**Table 4:** ROC curve results of inflammatory markers

Values	WBCC	NE%	NLR	CRP
Cutoff	7.3 * 10^9^/L	75.3%	8.2	10.0 mg/L
P value	< 0.001	< 0.001	< 0.001	< 0.001
AUC (95% CI)	0.659 (0.580–0.737)	0.708 (0.638–0.778)	0.761 (0.696–0.826)	0.790 (0.720–0.859)

*AUC* area under curve, *CI* confidence interval

## Discussion

Groin hernia is a common clinical disease, and conservative treatment may be considered for children under 2 years old. Once diagnosed with groin hernia in adult patients, elective surgical treatment should be considered [19]. When the contents of the abdominal cavity enter the hernia sac and cannot be returned on their own, emergency treatment is required. Clinical doctors can choose between manual reduction, open surgery, or laparoscopic surgery for treatment [19–21]. We diagnose the severity of incarcerated groin hernia through inflammatory markers to assist clinical doctors in making decisions.

A meta-analysis report suggests that gender is a high-risk factor for resection of incarcerated groin hernia [2]. And previous research reports also support gender as a high-risk factor for intestinal resection [17, 22]. However, it was found in this study that gender is not a high risk factor for resection of incarcerated groin hernia. Previous literature reports have also reached similar conclusions as this study [4, 5, 13].

This study found that the type of hernia is a risk factor for intestinal resection of incarcerated groin hernia. This supports some previous literature reports [2, 5, 17, 22]. The research reports of Turan et al. and Xie et al. refute this conclusion [4, 13]. It is worth mentioning that the research report by Xie et al. only reported 95 cases [13]. Turan, U et al. reported 132 cases, of which only 19 were female [4]. This may have caused some bias in their analysis. At the same time, we found that the incidence rate of direct hernia in incarcerated groin hernia is very low. Similar situations have also existed in previous literature [5, 13, 17]. Interestingly, we found that in Chen, Peng et al.’s study, there were 167 male cases (167/323, 51.7%) in the non-intestinal resection group and 168 cases (168/323, 52.0%) in the non-intestinal resection group with indirect hernia [17]. In Eyvaz et al.’s study, there were 75 male cases (75/129, 58.3%) in the non-intestinal resection group and 78 cases (78/129, 60.6%) in the non-intestinal resection group with indirect hernia [22]. This is significantly higher than the results in this study, where there were 75 male cases (75/203, 36.9%) in the non-intestinal resection group and 70 cases (70/203, 34.5%) in the non-intestinal resection group with indirect hernia. Considering that we have come to the opposite conclusion regarding whether gender is a high-risk factor. We have conducted further thinking. A femoral hernia is located in the area of the femoral canal below the inguinal ligament, and is a hernia formed when abdominal contents protrude into the femoral canal through the femoral ring. The space of the femoral tube is narrow, and the author found in clinical work that incarcerated femoral hernia is more difficult to manually reduce compared to incarcerated groin hernia. The research report by Zhou et al. pointed out that the proportion of incarcerated femoral hernias in female is higher than that in male [18]. We speculate that the reason for this difference is that we performed manual reduction on patients with incarcerated groin hernia. Manual reduction is an effective treatment measure, suitable for patients who have the intention of elective surgery or have poor general conditions. Of course, in our institution, we are cautious when performing manual reduction procedures on patients. Once the manual reduction fails, we will immediately prepare emergency surgery for the patient.

The age of the intestinal resection group was significantly higher than that of the non-intestinal resection group, which showed statistical differences. This indicates that advanced age is a high-risk factor affecting intestinal resection. Previous literature reports also support this viewpoint [2, 3]. We speculate that this may be related to the delayed diagnosis and treatment of elderly patients, and perhaps strengthening education can improve this situation. At the same time, this study found that the duration of incarceration of the intestinal resection group was significantly higher than that of the non-intestinal resection group, which had statistical differences. This indicates that duration of incarceration is also a high-risk factor for intestinal resection, which supports the conclusions of previous studies [2, 8, 23, 24]. This should be considered as the prolonged duration of incarceration will exacerbate intestinal ischemia and hypoxia, leading to intestinal necrosis. It is interesting that both the intestinal resection group and the non-intestinal resection group have a very large time span of entrapment. This indicates that we cannot determine whether a patient has developed intestinal necrosis based on a single time. The entrapment time of incarcerated groin hernia is not an absolute contraindication for manual reduction. We speculate that this may be due to some patients being unable to accurately express the time of occurrence of impaction. Or the degree of compression of the hernia contents by the hernia ring may vary.

This study found that hyponatremia is also a high-risk factor for incarcerated groin hernia resection surgery. This is similar to the results of Keeley, JA et al. [25]. We speculate that this may be related to intestinal obstruction caused by incarcerated groin hernia, leading to vomiting and hyponatremia. Therefore, we need to accelerate the treatment of incarcerated groin hernia patients with hyponatremia.

We divided them into two groups based on the time from admission to surgery, and found no statistical difference between the two groups. This seems to indicate that a short-term extension after admission will not worsen the severity of incarcerated groin hernia. The research results of Knewitz et al. are similar to this [26]. Of course, we still believe that the faster the treatment of incarcerated groin hernia, the better, especially for patients with short incarcerated time.

In this study, the cutoff value of NLR was 8.2. The research report by Chen et al. indicates that the NLR cutoff value is 6.5 [17]. Zhou et al. also believe that the cutoff value is 6.5 [5]. The cutoff value of Xie et al.’s report is 11.5 [13]. Turan et al.’s study showed that the cutoff value of NLR is 6.66 [4]. This means that the current reference values for NLR are controversial. Therefore, based on the ROC curve, this study selected a cutoff value of 8.2 for NLR. Meanwhile, WBCC, NE%, and CRP are common inflammatory markers, and we use the indicators from the blood analyzer as our reference values. In categorical variable analysis, we found that the proportion of increased inflammatory marker values in the intestinal resection group was significantly higher than that in the non-intestinal resection group. And they have statistical differences. In this study, the inflammatory marker values in the intestinal resection group were significantly higher than those in the non-intestinal resection group. There is a statistical difference between the two groups. This means that an increase in WBCC, NE%, CRP, and NLR values is a high-risk factor for intestinal resection of incarcerated groin hernia. And this confirms some previous literature reports [2, 4, 5, 13, 17, 22].

This study investigated the value of combining inflammatory markers in the diagnosis of intestinal necrosis in incarcerated groin hernia. When WBCC, NE%, and CRP values all normal, there were 3 (3/48, 6.3%) patients in the intestinal resection group and 45 (45/48, 93.7%) patients in the non-intestinal resection group. When WBCC, NE%, and CRP values all increased, there were 30 (30/33, 90.9%) patients in the intestinal resection group and 3 (3/33, 9.1%) patients in the non-intestinal resection group. We also included NLR in the analysis. When WBCC, NE%, NLR, and CRP values all normal, there were 2 patients (2/47, 4.3%) in the intestinal resection group and 45 patients (45/47, 95.7%) in the non-intestinal resection group. When WBCC, NE%, NLR, and CRP values all increased, there were 27 patients (27/28, 96.4%) in the intestinal resection group and 1 patient (1/28, 3.6%) in the non-intestinal resection group. This indicates that the value of combining inflammatory markers in the diagnosis of incarcerated groin hernia and strangulated groin hernia is extremely high. However, this cannot fully predict intestinal necrosis in incarcerated groin hernia. This supports Xie et al.’s research that there are currently no biomarkers that can fully predict intestinal necrosis in incarcerated groin hernia [13]. The use of laparoscopic techniques to treat incarcerated groin hernia has been proven to be safe and feasible [20, 21], and early identification of intestinal necrosis in incarcerated groin hernia can help surgeons choose surgical methods.

The AUC values of NLR and CRP were higher than those of WBCC and NE%, indicating that NLR and CRP have higher predictive value for intestinal resection of incarcerated groin hernia than WBCC and NE%. Xie et al.’s research report found that when NLR ≥ 11.5, the value of predicting strangulated groin hernia is higher compared to WBCC and neutrophil count [13]. Chen et al. stated that when NLR > 6.5, the value of predicting strangulated groin hernia is higher than WBCC and neutrophil count [17]. We look forward to more research to demonstrate their diagnostic value.

When analyzing the data in this article, some limitations need to be considered. Firstly, as a single center retrospective study, our data is sourced from medical records and may have errors. Secondly, the amount of data in this study is not very large, which may bring some limitations to our analysis. In addition, CRP values less than or equal to 10.0 mg/L in our center are considered 10.0 mg/L, which may introduce bias to our analysis.

## Conclusions

Elevated WBCC, NE%, NLR, or CRP values are high-risk factors for intestinal resection of incarcerated groin hernia. The combination of inflammatory markers is highly effective in predicting the severity of incarcerated groin hernia. The severity of incarcerated groin hernia is related to the duration of symptoms, but short-term delay after admission does not seem to exacerbate the severity of incarcerated groin hernia. NLR and CRP predict strangulated groin hernia better than WBCC and NE%.

## Data Availability

The datasets generated and/or analyzed during this study are not publicly available due to patient privacy and ethical restrictions but are available from the corresponding author on reasonable request, subject to approval by the relevant ethics committee.

## Abbreviations

WBCC: Preoperative white blood cell count
NE%: Percentage of neutrophils
CRP: C-reactive protein
NLR: Neutrophil/lymphocyte ratio
serum Sodium-ion: Serum Na^+^
AUC: The area under the curve
CI: Confidence interval
